# Duplex, extraction-free, HiFi-LAMP assay for point-of-care detection of *Chlamydia trachomatis* and *Neisseria gonorrhoeae*

**DOI:** 10.1128/spectrum.03416-25

**Published:** 2026-07-24

**Authors:** Zhengfang Wang, Yongjuan Zhao, Yuhua Shi, Shangwen Song, Yingkui Cao, Yumeng Pan, Yang Yang, Yi-Qun Kuang, Chiyu Zhang, Yu-Ye Li

**Affiliations:** 1 Department of Dermatology and Venereology, First Aﬃliated Hospital of Kunming Medical University 71240, Kunming, Yunnan, People's Republic of China; 2 Shanghai Public Health Clinical Center, Fudan University 12478, Shanghai, People's Republic of China; 3 Yunnan Center for Disease Control and Prevention 590170, Kunming, Yunnan, People's Republic of China; 4 Research Center for Clinical Medicine, First Aﬃliated Hospital of Kunming Medical University 71240, Kunming, Yunnan, People's Republic of China; Institut National de Santé Publique du Québec, Sainte-Anne-de-Bellevue, Québec, Canada

**Keywords:** *Chlamydia trachomatis*, *Neisseria gonorrhoeae*, HiFi-LAMP, urine, genital secretion, extraction-free, point-of-care testing

## Abstract

*Chlamydia trachomatis* (*C. trachomatis*) and *Neisseria gonorrhoeae* (*N. gonorrhoeae*) are common sexually transmitted pathogens. Although frequently asymptomatic, they can cause severe reproductive complications (e.g., epididymitis, salpingitis, pelvic inflammatory disease, and infertility) if untreated. We developed a duplex high-fidelity loop-mediated isothermal amplification (HiFi-LAMP) assay for simultaneous detection of *C. trachomatis* and *N. gonorrhoeae*. The assay demonstrated high sensitivity and specificity, with limits of detection (LODs) of 358 and 30 copies per 25 μL reaction for *C. trachomatis* and *N. gonorrhoeae*, respectively. The performance of the assays was evaluated by comparing with the commercial singleplex qPCR assays using 156 paired urine and genital secretion samples. The assays have 100% specificity across all samples and achieved 100% sensitivity for the detection of *N. gonorrhoeae* in urine and *C. trachomatis* in genital secretion samples and 98.7% and 88.5% sensitivity for *N. gonorrhoeae* in genital secretions and *C. trachomatis* in urines, respectively. Extraction-free HiFi-LAMP assays directly using a small amount of simply treated urine were established and showed 87.9% sensitivity and 100% specificity for the detection of *N. gonorrhoeae* in 70 urine samples. The new HiFi-LAMP assays can detect all *C. trachomatis*- and/or *N. gonorrhoeae*-positive samples within 30 min, implying that the entire HiFi-LAMP assay (including sample process) can be completed within 40 min, significantly shorter than about 90 min by the qPCR assays. The new HiFi-LAMP assays provide a rapid, simple, and cost-effective point-of-care testing (POCT) tool for clinical detection and/or surveillance of *C. trachomatis* and *N. gonorrhoeae* in resource-limited settings.

**IMPORTANCE:**

Asymptomatic *C. trachomatis* and *N. gonorrhoeae* infections significantly increase the risk of silent transmission. *C. trachomatis* and *N. gonorrhoeae* were cocirculating in sexually active individuals. Of 156 individuals, we detected 7 (4.5%) individuals positive for both *C. trachomatis* and *N. gonorrhoeae*, implying an urgent need for rapid accurate and duplex POCT assay for timely detection of *C. trachomatis* and *N. gonorrhoeae*. The development of a duplex HiFi-LAMP assay enables the simultaneous detection of *C. trachomatis* and *N. gonorrhoeae* with high sensitivity and specificity. The assay offers high diagnostic concordance with commercial qPCR assays and has lower cost and shorter reaction time. Given the availability of various portable fluorescent thermal cyclers and/or isothermal fluorimeters, the development of extraction-free HiFi-LAMP assay directly using simply treated urine will lower the barriers of sexually active individuals to routine screening and facilitate clinical detection and surveillance of *C. trachomatis* and *N. gonorrhoeae* in resource-limited settings.

## INTRODUCTION

*Chlamydia trachomatis* (*C. trachomatis*) and *Neisseria gonorrhoeae* (*N. gonorrhoeae*) are among the most prevalent treatable sexually transmitted infections (STIs) globally. The World Health Organization (WHO) estimated 129 million new *C. trachomatis* infections and 82 million new *N. gonorrhoeae* infections in 2020 (1). The infection rate of *C. trachomatis* is 2.9% in the general population (2) and up to 20% in patients with pelvic inflammatory disease (PID) (3). Symptomatic *C. trachomatis* infection manifests predominantly as urethritis in males and cervicitis in females. In symptomatic *N. gonorrhoeae* infection, males typically present with dysuria and purulent urethral discharge, whereas females commonly exhibit vaginal discharge and pruritus. Despite these typical clinical manifestations, infections caused by *C. trachomatis* and *N. gonorrhoeae* are often clinically silent, which poses a major challenge. Epidemiological and clinical studies demonstrated that over half of such infections lack obvious clinical symptoms (4–7). Untreated *C. trachomatis* and/or *N. gonorrhoeae* infections can lead to severe and devastating complications (8). Approximately 60.7% of *C. trachomatis* and 53.3% of *N. gonorrhoeae* cases in women appeared to be asymptomatic, and persistent *C. trachomatis* and/or *N. gonorrhoeae* infections in women often ascend to the upper reproductive tract, causing PID, ectopic pregnancy, and infertility (9, 10). Furthermore, untreated *C. trachomatis* and/or *N. gonorrhoeae* infections increase human susceptibility to HIV infection (11, 12), which exacerbates disease progression, accelerates the global spread of sexually transmitted diseases, and imposes significant societal and economic burdens.

To mitigate the health risks caused by *C. trachomatis* and/or *N. gonorrhoeae* infections, the U.S. Centers for Disease Control and Prevention (CDC; https://www.cdc.gov/std/treatment-guidelines/screening-recommendations.htm) recommend annual routine screening for *C. trachomatis* and *N. gonorrhoeae* in sexually active individuals (13). Previous studies indicated that sexual health education for sexually active individuals increased screening rates for *C. trachomatis* and *N. gonorrhoeae*, thereby reducing the incidence of infections and PID (14, 15). Fast, simple, and sensitive point-of-care testing (POCT) assays are urgently needed to facilitate the screening of *C. trachomatis* and *N. gonorrhoeae* infections.

Traditional diagnostic methods, such as microscopy, culture, and immunological assays, suffer from sample type restrictions, low sensitivity, and long turnaround time, which limit their wide use in universal screening for *C. trachomatis* and *N. gonorrhoeae* (16). Consequently, nucleic acid amplification tests (NAATs) have been widely considered due to their high sensitivity (17, 18). In particular, quantitative PCR (qPCR) was often used as a gold standard for the molecular diagnosis of various infectious diseases due to high sensitivity, specificity, and multiplex detection capability (19). However, high dependence on sophisticated instruments and technicians, as well as a longer turnaround time (about 1.5 h), limits its potential for rapid detection of *C. trachomatis* and *N. gonorrhoeae* in resource-limited settings (20). Therefore, the development of an affordable, highly sensitive and specific, user-friendly POC diagnostic assay is a high priority for the prevention and control of *C. trachomatis* and *N. gonorrhoeae*.

Loop-mediated isothermal amplification (LAMP) offers significant advantages for POC diagnostics due to its high sensitivity, minimal equipment requirements, simplicity, and rapid turnaround time (21). We previously developed a HiFi-LAMP method to well overcome the inherent drawbacks of the conventional LAMP assays, such as non-specific amplification signal and the lack of multiplex detection capability (22, 23). The success of the HiFi-LAMP method is attributed to the use of an HFman probe that can be cleaved by the 3′–5′ exonuclease activity of high-fidelity DNA polymerases to release fluorescent signal (24, 25). In this study, we developed a duplex HiFi-LAMP assay and its extraction-free version for rapidly detecting *C. trachomatis* and *N. gonorrhoeae* and demonstrated its high potential for *C. trachomatis* and *N. gonorrhoeae* screening in resource-limited settings.

## MATERIALS AND METHODS

### Preparation of primers, probes, and DNA standards

Three sets of LAMP primers targeting to *ompA* and *gyrA* genes of *C. trachomatis* and three sets targeting to *penA*, *orf1*, and *porA* genes of *N. gonorrhoeae* were described previously (Table S1). Each of the LAMP primer sets includes three pairs of primers: forward/backward outer primers (F3/B3), forward/backward inner primers (FIP/BIP), and forward/backward loop primers (LF/LB), except the primer set CT3 that lacks F3 and LF (Table S1). The HFman probe was designed by labeling the fluorophore and quencher groups at the 3′ and 5′ ends of the loop primer, respectively. All primers and probes were synthesized by Sangon Biotech (Shanghai, China).

A pUC57 plasmid containing *ompA* (GeneBank: JX564246.1) and *gyrA* (CP106985.1) genes of *C. trachomatis* and another containing *penA* (PQ000243.1), *orf1* (M84113.1), and *porA* (AJ223447.1) genes of *N. gonorrhoeae* were separately synthesized by Sangon Biotech (Shanghai, China) and used as DNA standards. DNA concentration was measured using a Qubit 4.0 Fluorometer (Thermo Fisher Scientific, USA). The copy number of DNA standard was calculated using the following formula: dsDNA copies/µL = 9.12 × 10¹¹ × DNA concentration (ng/μL)/target fragment length.

### Selection of the optimal LAMP primer set

Conventional LAMP assay with fluorescent dye SYTO-9 was used to screen the optimal LAMP primer set for the detection of *C. trachomatis* and *N. gonorrhoeae* as previously described (26). In brief, all three primer sets for each pathogen were separately subjected to the conventional LAMP assay with the same template input. The primer set that yields the earliest amplification curve (with the lowest time threshold, Tt) in the LAMP reaction indicates the highest amplification efficiency and will be used as the primer candidate for the development of the HiFi-LAMP assay. A standard 25 µL LAMP reaction contains 8 U Bst 4.0 DNA/RNA polymerase (Haigene, China), 1 × isothermal amplification buffer, 1.4 mM dNTP mix, 1 mM SYTO 9 Green Fluorescent Nucleic Acid Stain (Invitrogen, USA), 6 mM MgSO₄, 0.1 µM F3 and B3 each, 1 µM FIP and BIP each, 0.6 µM LF and LB each. Reactions were run on the CFX 96 Touch Real-Time PCR Detection System (Bio-Rad, USA) at 63°C for 50 min, with fluorescence signals to be collected per minute in the SYBR channel.

### Establishment of the singleplex and duplex HiFi-LAMP assays

A standard singleplex HiFi-LAMP reaction was established based on the conventional LAMP reaction system by replacing SYTO 9 with 0.3 µM HFman probe and halving the concentration of the loop primer that shares identical sequence to the probe from 0.6 µM to 0.3 µM. In the duplex HiFi-LAMP reaction, the concentrations of all primers and probes for each target were further halved compared to the singleplex HiFi-LAMP assay. The HiFi-LAMP assay was carried out using the CFX 96 Touch Real-Time PCR Detection System (Bio-Rad, USA) at 63°C for 50 min, with fluorescence signals to be collected per minute in the FAM and/or CY5 channels.

### Sensitivity, specificity, and the limit of detection of the HiFi-LAMP assay

The DNA standards of *C. trachomatis* and *N. gonorrhoeae* were 10-fold serially diluted from 10,000 copies/µL to 1 copy/µL to assess the sensitivity of the HiFi-LAMP assays. The specificities of the HiFi-LAMP assays were evaluated using 3 common sexually transmitted pathogens (*Ureaplasma urealyticum* [UU], *Herpes simplex virus type 2* [HSV-2], *Mycoplasma hominis* [MH]) and 11 common respiratory pathogens (*Viridans streptococci* [VS], *Influenza virus* [IV], *Pseudomonas aeruginosa*, *Klebsiella pneumoniae*, *Group B streptococcus*, *Bacillus Calmette-Guérin* [BCG], *Streptococcus pyogenes*, *Candida parapsilosis*, *Streptococcus pneumoniae*, *Candida albicans*, and *Staphylococcus aureus*). Among these pathogens, BCG was a standard strain purchased from Gene-Optimal (Shanghai, China), HSV-2 was derived from positive clinical sample by a qPCR assay, and the remaining pathogens were obtained from clinical specimens confirmed positive by culture-based methods. Nucleic acids were extracted from standard strains or clinical samples using a commercial DNA/RNA extraction kit that is based on magnetic bead technology (TIANGEN, Beijing, China).

To determine the LODs of the assays, 5-fold serially diluted DNA standards from 3,000 to 5 copies were used in the 25 µL HiFi-LAMP reaction with 32 replicates for each concentration. The LOD was derived as the concentration yielding 95% positive results using a probit regression analysis implemented in SPSS 17.0 software.

### Clinical evaluation of the HiFi-LAMP assay

A total of 156 paired urine and genital secretion samples previously collected from patients who visited the First Aﬃliated Hospital of Kunming Medical University were used to assess the performance of the HiFi-LAMP assay for *C. trachomatis* and *N. gonorrhoeae* detection (Table S2). The use of the clinical samples was approved by the ethics committee of the First Afﬁliated Hospital of Kunming Medical University (No. 2022-L-239) with waiver of informed consents. DNA was extracted from 1 mL of urine or genital secretion samples in a biosafety level 2 laboratory using the Bacterial Genomic DNA Extraction Kit (TIANGEN, Beijing, China) that is based on spin column-based method. Two commercial singleplex qPCR assays for *C. trachomatis* and *N. gonorrhoeae* (Sansure Biotech, Changsha, China) were used as gold standard to evaluate the HiFi-LAMP assays. According to the manufacturer’s instructions, the sample with a cycle threshold (Ct) value of <38 in the qPCR assay was defined as positive for *C. trachomatis* and/or *N. gonorrhoeae*.

### Urine sample processing for extraction-free HiFi-LAMP assay

Raw urine was mixed with a commercial nucleic acid release buffer (Sansure Biotech, Changsha, China) at a 1:1 ratio according to the manufacturer’s instruction. The buffer mainly consists of potassium chloride, absolute ethanol, Savantine (C₅₃H₉₃N₇O₁₃), sodium dodecyl sulfate (SDS), and sterilized purified water. After incubation at room temperature for 10 min, treated urine can be directly used in the HiFi-LAMP assay. Because urea in urine can inhibit nucleic acid amplification (27), we further diluted the treated urine samples with enzyme-free water at ratios ranging from 1:1 to 1:6 to determine the optimal dilution with minimal inhibitory effect of urine on the HiFi-LAMP amplification.

### Statistical analysis

All statistical analyses were performed using SPSS 17.0 or GraphPad Prism 10.0. The correlation between Ct and Tt values was evaluated using Pearson correlation and Linear regression analyses. The 95% confidence interval (CI) was calculated using the Wilson score method. Comparison of detection rates between different specimen types was performed using Chi-square test. A *P* value < 0.05 was considered statistically significant. Each experiment was performed in duplicate, and all experiments were repeated at least twice.

## RESULTS

### Screening of primers and probes for *C. trachomatis* and *N. gonorrhoeae*

Three sets of LAMP primers for each pathogen were subjected to the screening of the optimal primer set for the detection of *C. trachomatis* (CT) and *N. gonorrhoeae* (NG) (Table S1). Primer sets CT1 and CT2 demonstrated the lower Tt values than CT3 for *C. trachomatis* detection (Fig. S1A). Because CT2-LF corresponds to a more conserved genomic region than CT1-LF (Fig. S2), the primer set CT2 was selected preferentially for the development of HiFi-LAMP assay. For *N. gonorrhoeae* detection, the primer set NG2 was selected since it exhibited the fastest amplification with the lowest Tt value (Fig. S1B). Although the two selected primer sets (CT2 and NG2) exhibited the highest amplification efficiency, they yielded non-specific amplification signals in reactions without templates (Fig. S1). To eliminate non-specific amplification signal and enable multiplex detection, two HFman probes were designed for developing HiFi-LAMP assay based on the LFs of the primer sets CT2 and NG2 (Table S1).

To test the specificity of the HiFi-LAMP assay, non-template control (NTC) was performed in 16 replicates. Non-specific amplification signals were observed in the HiFi-LAMP assays for both *C. trachomatis* and *N. gonorrhoeae* (Fig. S3A and B), implying low specificity. To improve the specificity, we further designed several loop primers and HFman probes for the HiFi-LAMP assays (Table S1). After several rounds of screening with various combination of primers and HFman probes (Fig. S3C and D), HFman-LB probe for *C. trachomatis* and the combination of HFman-LF1 probe with LF1 and LB2 loop primers for *N. gonorrhoeae* achieved the best specificity (without non-specific amplification signals in NTC) (Fig. S3E through L) and were used to establish the singleplex and duplex HiFi-LAMP assays for the detection of *C. trachomatis* and/or *N. gonorrhoeae*.

### Sensitivity and specificity of the HiFi-LAMP assays for the detection of *C. trachomatis* and *N. gonorrhoeae*

**Fig 1:**
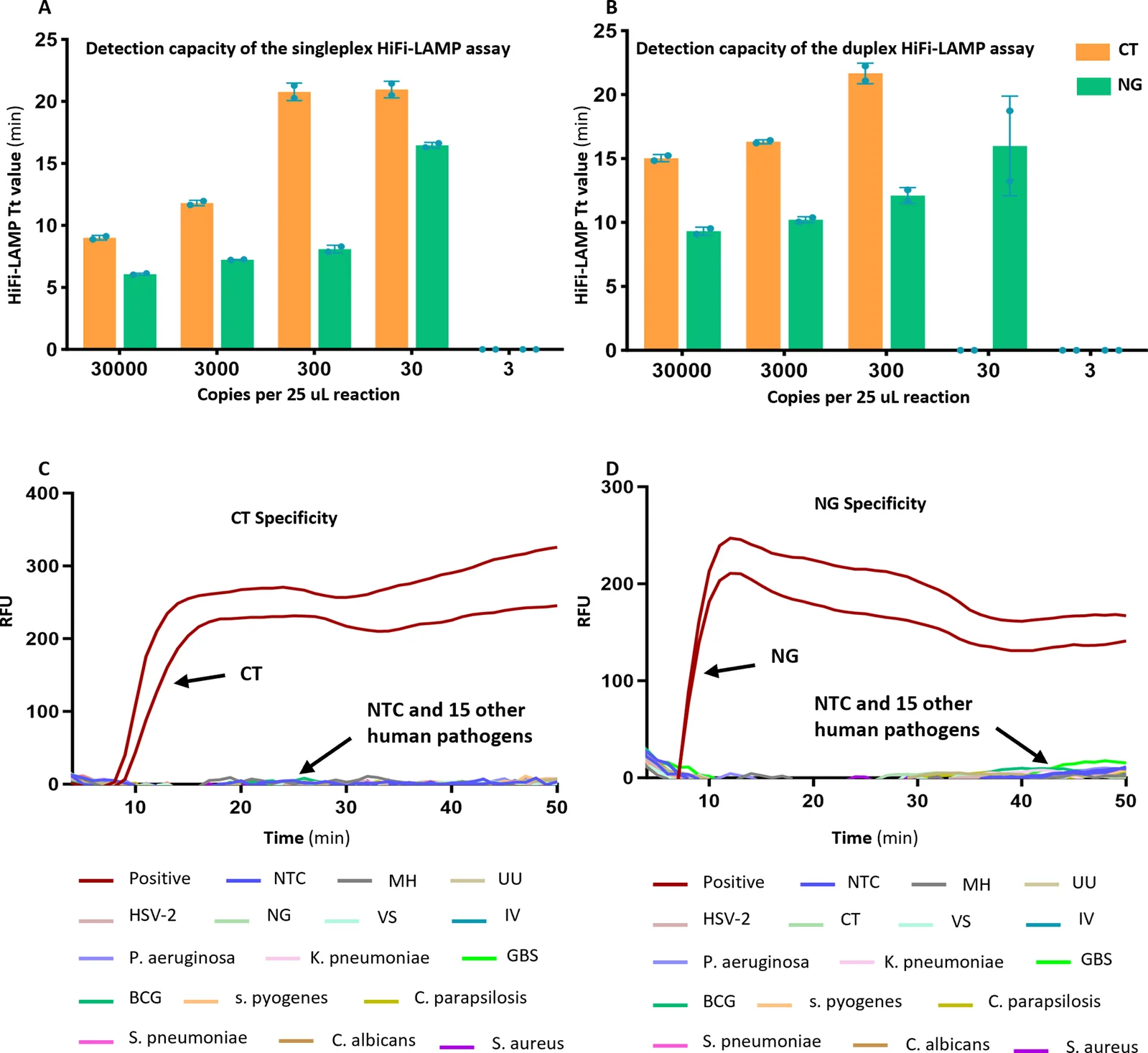
Sensitivity and specificity of the HiFi-LAMP assay for *C. trachomatis* and *N. gonorrhoeae*. (**A**) Sensitivities of the singleplex HiFi-LAMP assays. (**B**) Sensitivity of the duplex HiFi-LAMP assay. (**C and D**) Specificities of the HiFi-LAMP assays. The data (Tt values) are shown as the mean ± SD. CT, *C. trachomatis*; NG, *N. gonorrhoeae*. MH, *Mycoplasma hominis*; UU, *Ureaplasma urealyticum*; HSV-2, *Herpes simplex virus* type 2; VS,* Viridans* streptococci; IV, *Influenza virus*; *P. aeruginosa, Pseudomonas aeruginosa*; *K. pneumoniae, Klebsiella pneumoniae*; GBS, *Group B streptococcus*; BCG, *Bacillus Calmette-Guérin*; *S. pyogenes*, *Streptococcus pyogenes*; *C. parapsilosis, Candida parapsilosis*; *S. pneumoniae, Streptococcus pneumoniae*; *C. albicans*, *Candida albicans*; *S. aureus*, *Staphylococcus aureus*.

The singleplex HiFi-LAMP assay can stably detect 30 copies of *C. trachomatis* or *N. gonorrhoeae* per 25 μL reaction (Fig. 1A). Compared with the singleplex assays, the duplex HiFi-LAMP assay showed identical sensitivity for NG but lower sensitivity for *C. trachomatis* (300 copies per 25 μL reaction) (Fig. 1B). The LODs of the singleplex HiFi-LAMP assays were determined to be 358 and 30 copies per 25 µL reaction for *C. trachomatis* and *N. gonorrhoeae*, respectively (Table 1).

**TABLE 1:** The LODs of the singleplex HiFi-LAMP assays for the detection of *C. trachomatis* and *N. gonorrhoeae*^*a*^

Template input (copies per 25 µL reaction）	CT (Positive/total)	NG (Positive/total)
3,000	32/32	32/32
600	31/32	32/32
120	31/32	32/32
24	29/32	28/32
5	11/32	13/32
LOD	358	30

^*a*^ CT, *C. trachomatis*; NG, *N. gonorrhoeae*.

The speciﬁcities of the HiFi-LAMP assays were evaluated using 3 common sexually transmitted pathogens and 11 common respiratory pathogens. No amplification signal was observed for the 14 above-mentioned pathogens (Fig. 1C and D). Furthermore, no cross-reaction was observed between *C. trachomatis* and *N. gonorrhoeae*. These results indicate that the HiFi-LAMP assays have excellent specificity.

### Detection performance of the HiFi-LAMP assays for *C. trachomatis* and *N. gonorrhoeae* in clinical samples

**Fig 2:**
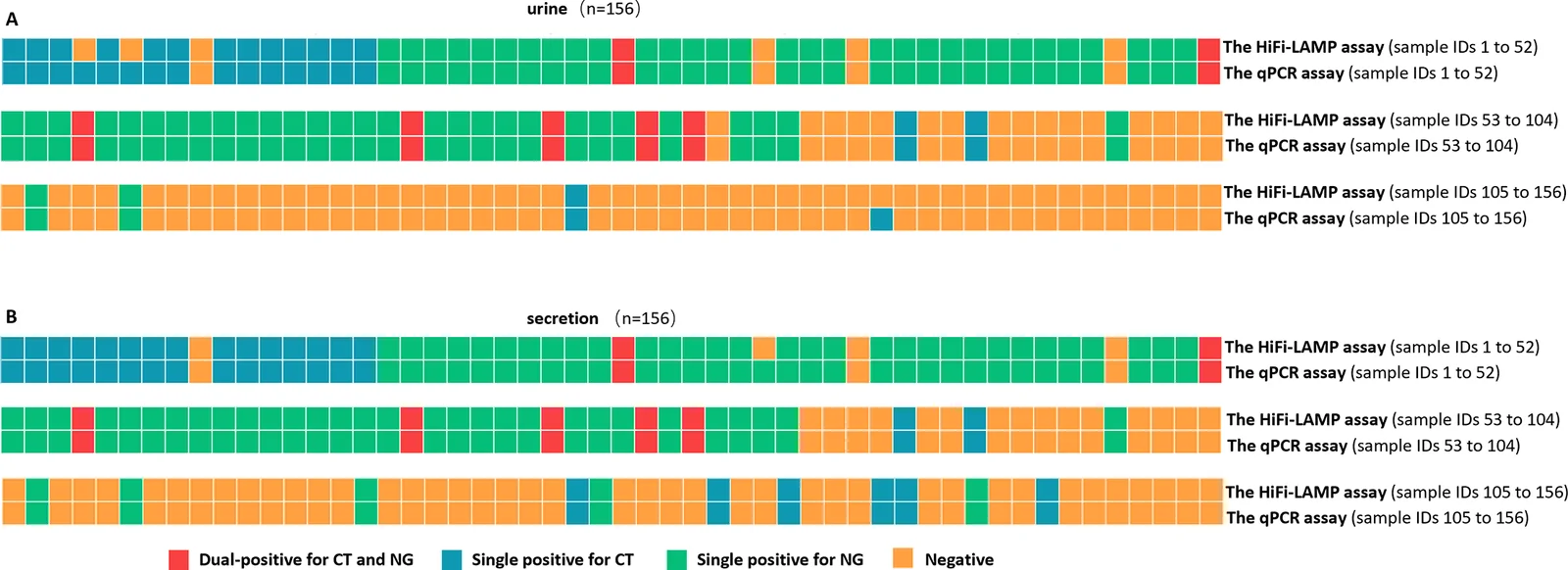
Detection of *C. trachomatis* and *N. gonorrhoeae* in 156 paired urine and genital secretion samples by commercial qPCR and the new HiFi-LAMP assays. CT, *C. trachomatis*; NG, *N. gonorrhoeae*.

**Fig 3:**
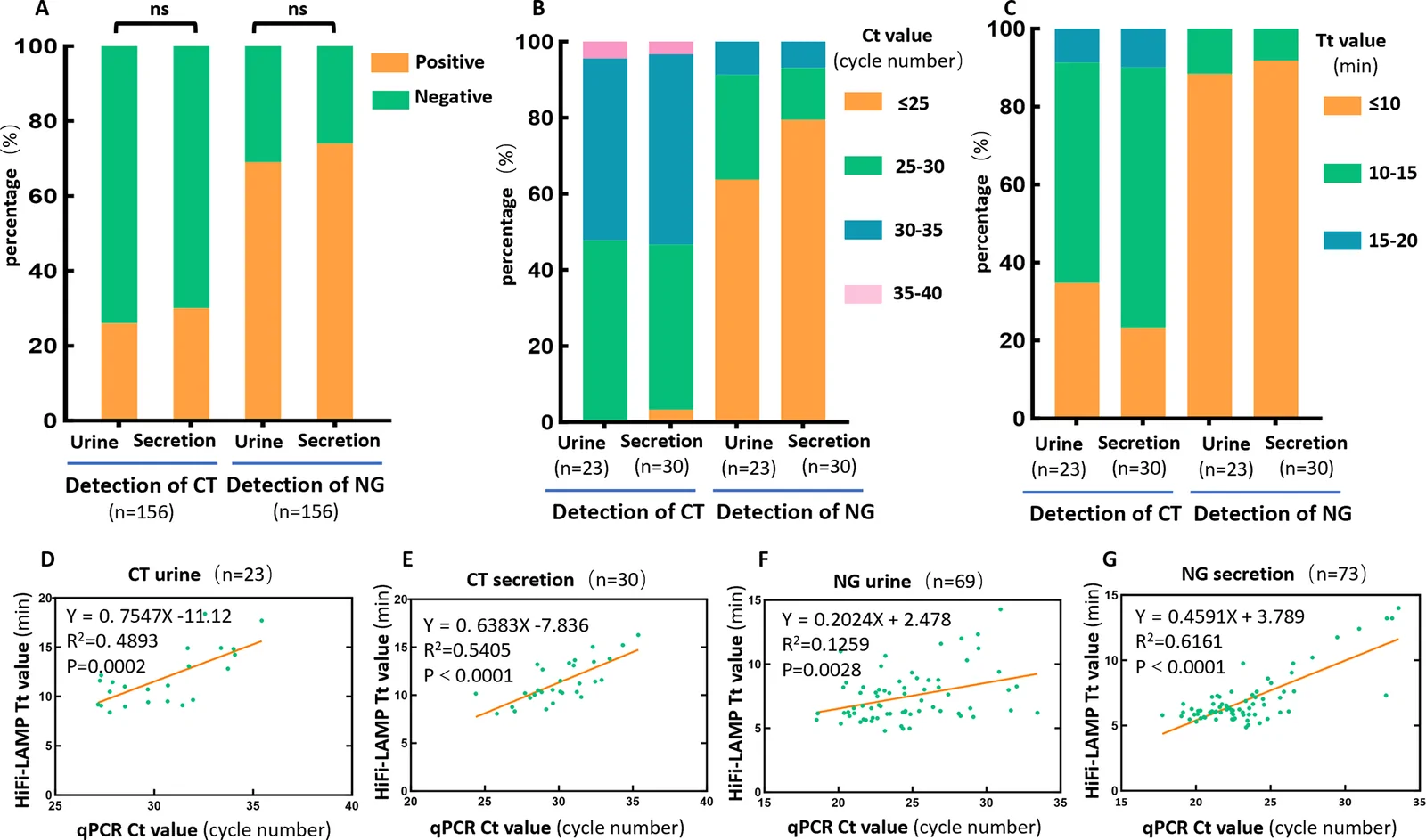
Comparison of detection of *C. trachomatis* and *N. gonorrhoeae* in 156 paired urine and genital secretion samples between commercial qPCR and the new HiFi-LAMP assays. (**A**) Detection rates of *C. trachomatis* and *N. gonorrhoeae* by the qPCR assays. (**B**) Distribution of Ct values of positive samples by the qPCR assays. (**C**) Distribution of Tt values of positive samples by the HiFi-LAMP assays. (**D–G**) Correlation analyses between the Ct values of the qPCR assays and the Tt values of the HiFi-LAMP assays. CT, *C. trachomatis*; NG, *N. gonorrhoeae*.

A total of 156 paired urine and genital secretion samples were used to evaluate the performance of the HiFi-LAMP assay. Two commercial qPCR kits were used as the gold standards for the detection of *C. trachomatis* and *N. gonorrhoeae*. Among the 156 involved individuals, 146 (93.6%) were male, most were in the 20–40 age (97/156, 62.2%), and the most frequent education level was a bachelor’s degree (72/156, 46.2%) (Table S2). The qPCR assays detected 26 (16.7%, 95% CI: 10.8%–22.6%) and 30 (19.2%, 95% CI: 13.0%–25.5%) *C. trachomatis* positives, and 69 (44.2%, 95% CI: 36.4%–52.1%) and 74 (47.4%, 95% CI: 39.5%–55.4%) *N. gonorrhoeae* positives in 156 paired urine and genital secretion samples, respectively (Fig. 2 and Table 2). Seven paired urine and secretion samples were detected positive for both *C. trachomatis* and *N. gonorrhoeae*, suggesting a coinfection rate of 4.5% (95% CI: 1.2%–7.8%). The detection rates of both pathogens were slightly higher in genital secretions than in urines (Fig. 3A), suggesting that genital secretion is more suitable for the detection of *C. trachomatis* and *N. gonorrhoeae* than urine. In addition, positive genital secretion samples had substantially lower Ct values than positive urine samples (Fig. S4), suggesting higher bacterial load in genital secretions than urines.

**TABLE 2:** Detection of *C. trachomatis* and *N. gonorrhoeae* in 156 paired clinical samples by both the HiFi-LAMP and qPCR assays^*a*^

Methods	The Sansure Biotech qPCR assay												
		CT (urine)			CT (genital secretion)			NG (urine)			NG (genital secretion)		
		Pos.	Neg.	Total	Pos.	Neg.	Total	Pos.	Neg.	Total	Pos.	Neg.	Total
The singleplex HiFi-LAMP assay	Pos.	23	0	23	30	0	30	69	0	69	73	0	73
	Neg.	3	130	133	0	126	126	0	87	87	1	82	83
	Total	26	130	156	30	126	156	69	87	156	74	82	156
Sensitivity		(23/26) 88.5%			(30/30) 100%			(69/69) 100%			(73/74) 98.7%		
Specificity		(130/130) 100%			(126/126) 100%			(87/87) 100%			(82/82) 100%		
Consistency		(153/156) 98.1%			(156/156) 100%			(156/156) 100%			(155/156) 99.4%		

^*a*^ Pos., positive; Neg., negative; CT, *C. trachomatis*; NG, *N. gonorrhoeae*.

The HiFi-LAMP assay detected 30 (19.2%, 95% CI: 13.0%–25.5%) *C. trachomatis* positives in genital secretions and 69 (44.2%, 95% CI: 36.4%–52.1%) *N. gonorrhoeae* positives in urine samples showing 100% sensitivity and 100% specificity, but 23 (14.7%, 95% CI: 9.1%–20.4%) *C. trachomatis* positives in urine samples, and 73 (46.8%, 95% CI: 38.9%–54.7%) *N. gonorrhoeae* positive in genital secretion samples, showing 88.5% and 98.7% sensitivities for *C. trachomatis* and *N. gonorrhoeae* in the two sample types, respectively (Fig. 2 and Table 2).

We compared the Ct values of the qPCR assays and Tt values of the HiFi-LAMP assays for the *C. trachomatis*- and *N. gonorrhoeae*-positive samples. In the qPCR assays, the majority of *C. trachomatis*-positive urine and genital secretion samples had Ct values of between 25 and 35 (22/23, 95.7%, 95% CI: 86.6%–104.7% and 28/30, 93.3%, 95% CI: 83.9%–102.8%), whereas the majority of the *N. gonorrhoeae*-positive sample had Ct values of less than 25 (44/69, 63.8%, 95% CI: 52.1%–75.4% and 58/73, 79.5%, 95% CI: 70.0%–89.0%) (Fig. 3B). This result indicates that the bacterial load of *N. gonorrhoeae* was substantially higher than that of *C. trachomatis* in these samples. In the HiFi-LAMP assays, about half of the *C. trachomatis*-positive samples (including urine and genital secretion) had Tt values of less than 10 min, whereas the proportion of *N. gonorrhoeae*-positive samples with Tt values of less than 10 min was 61/69 (88.4%, 95% CI: 80.7%–96.2%) and 67/73 (91.8%, 95% CI: 85.3%–98.2%) in urine and genital secretions, respectively (Fig. 3C), supporting a higher load of *N. gonorrhoeae* than *C. trachomatis*. In particular, all *C. trachomatis*- and *N. gonorrhoeae*-positive samples by the HiFi-LAMP assays had Tt values of less than 15 min except two urine (17.7 and 18.4 min) and three genital secretion samples (15.0, 15.2, and 16.3 min) for *C. trachomatis*. These results indicated that the HiFi-LAMP assays are very fast and can be completed within 20 min.

Correlation analyses showed that the Tt values of the HiFi-LAMP assays were significantly positively linearly correlated with the Ct values of the commercial qPCR assays for *C. trachomatis* (R2: 0.4893 and 0.540, respectively, both *P* < 0.0002) and *N. gonorrhoeae* (R2: 0.1259 and 0.6161, respectively, both *P* < 0.0028 ) in urine and genital secretion samples (Fig. 3D through G).

To assess the performance of the duplex HiFi-LAMP assay, 86 urine samples confirmed by the singleplex HiFi-LAMP assay were used, including 13 *C. trachomatis*-positive, 59 *N. gonorrhoeae*-positive, 7 *C. trachomatis*- and *N. gonorrhoeae*-dual positive (co-infection), and 7 negative samples (Table S3). All these samples, including seven co-infected samples, were correctly identified by the duplex HiFi-LAMP assay (Table S3 and Fig. S5), showing 100% concordance with the singleplex assays. These results demonstrated that the duplex HiFi-LAMP assay has excellent performance for the simultaneous detection of *C. trachomatis* and *N. gonorrhoeae*.

### Development and clinical validation of a urine extraction-free HiFi-LAMP assay

**Fig 4:**
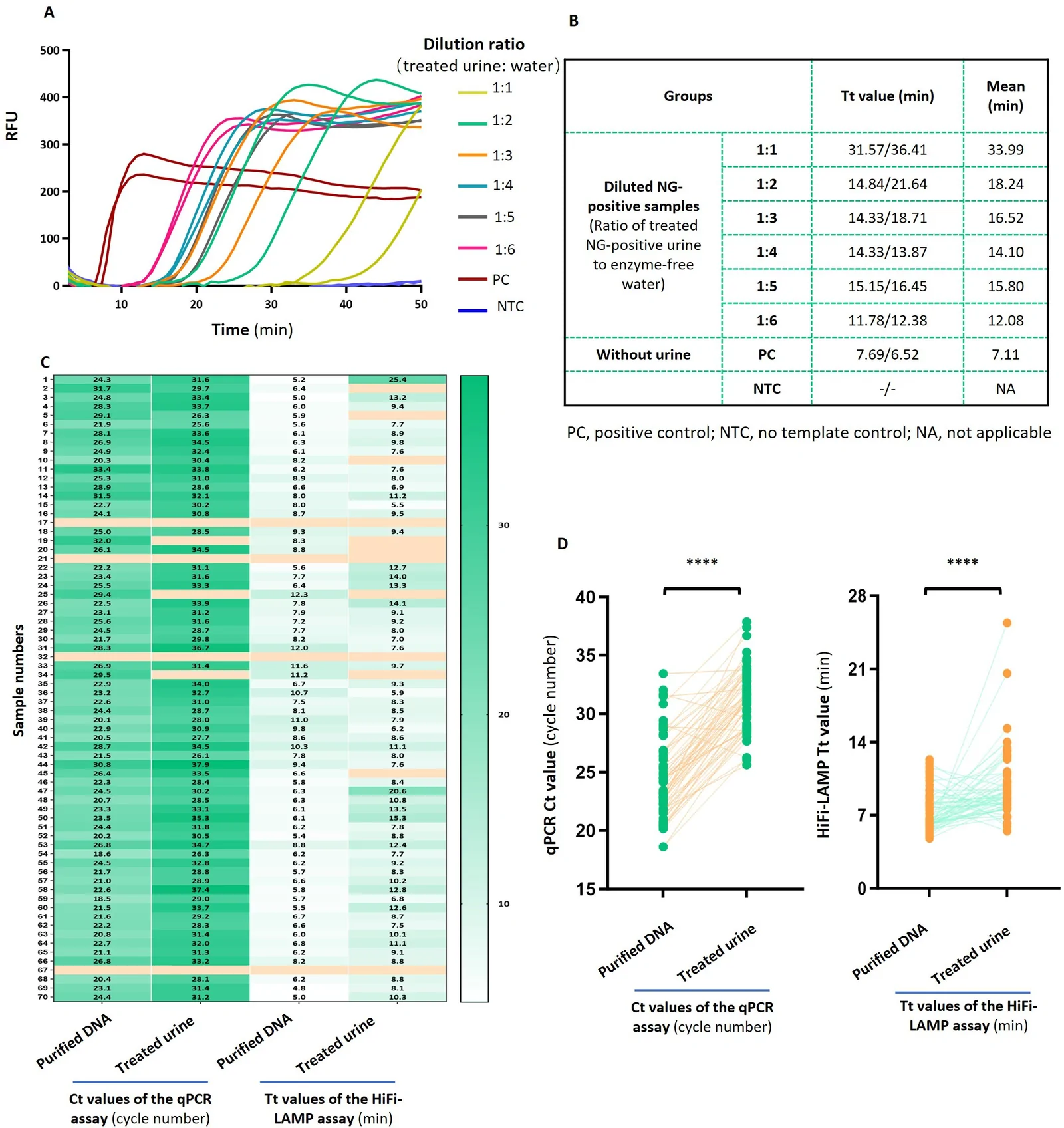
Development and clinical evaluation of the extraction-free HiFi-LAMP assay for the detection of *N. gonorrhoeae* in urine samples. (**A and B**) Optimization of urine processing strategies. (**C**) Heatmap of the qPCR assay Ct values and the HiFi-LAMP assay Tt values for 70 urine samples, using purified DNA and simply treated urine. (**D**) Comparisons of the qPCR Ct values and the HiFi-LAMP Tt values between the conventional version with purified DNA and the extraction-free version directly using urine. CT, *C. trachomatis*; NG, *N. gonorrhoeae*. ****P < 0.0001.

To develop an extraction-free HiFi-LAMP assay, urine was first lysed with an equal volume of a nucleic acid release buffer, followed by dilution with enzyme-free water at different ratios. The results showed that the fastest amplification with the lowest Tt value (mean Tt value: 12.1 min) was generated in the reactions with 1:6 diluted lysed urine (equivalent to 0.214 μL of raw urine) (Fig. 4A and B), implying minimum inhibition in amplification.

To evaluate the performance of the extraction-free HiFi-LAMP assay, 70 urine samples, including 66 *N. gonorrhoeae*-positive and 4 *N. gonorrhoeae*-negative samples, were tested. Of 66 positive samples, 63 (95.5%, 95% CI: 90.1%–100.6%) and 58 (87.9%, 95% CI: 79.8%–96.0%) were correctly identified by the extraction-free qPCR assay and HiFi-LAMP assay, respectively (Fig. 4C and Table 3). Furthermore, the extraction-free qPCR and HiFi-LAMP assays had significantly higher Ct (25.6–37.9) and Tt (5.5–25.4) values than their traditional versions with purified DNA (18.5–33.4 and 4.8–12.3, respectively) (Fig. 4D), implying relatively slower amplification. The slightly lower detection rates and higher Ct or Tt values by both extraction-free qPCR and HiFi-LAMP assays were mainly ascribed to extremely low sample input (about 0.214 μL raw urine) compared with their traditional assays with purified DNA (equivalent to 125 μL raw urine). Nevertheless, except for 2 samples with Tt values of 20.6 and 25.4, all positive samples had Tt values of less than 16, implying that the detection could be completed within 30 min. All four negative samples were detected negative by both extraction-free assays, showing 100% specificity. These results highlight the potential of the extraction-free HiFi-LAMP assay for rapid POCT detection of *N. gonorrhoeae* and *C. trachomatis*.

**TABLE 3:** Performance of the extraction-free HiFi-LAMP and qPCR assays for detecting *N. gonorrhoeae* in 70 urine samples^*a*^

Methods	Total	TP	TN	FP	FN	SE	SP
Extraction-free HiFi-LAMP assay	70	58	4	0	8	87.9%	100%
Extraction-free qPCR assay	70	63	4	0	3	95.5%	100%

^*a*^ TP, true positive; TN, true negative; FP, false positive; FN, false negative; SE, sensitivity; SP, specificity.

## DISCUSSION

*C. trachomatis* and *N. gonorrhoeae* infections represent two of the most common bacterial STIs worldwide. In spite of more than half of asymptomatic infections, untreated *C. trachomatis* and/or *N. gonorrhoeae* infections can lead to severe long-term complications, such as PID, epididymitis, and infertility (8, 28, 29). Asymptomatic individuals may act as an important silent reservoir, accelerating the spread and transmission of *C. trachomatis* and *N. gonorrhoeae*. Therefore, the WHO recommended routine screening for *C. trachomatis* and *N. gonorrhoeae* for populations at risk (e.g., sexually active women, pregnant women, and men who have sex with men [MSM]) (2025 guideline: https://www.who.int/publications/i/item/9789240104907). The U.S. CDC STI treatment guidelines strongly recommended routine screening for *C. trachomatis* and *N. gonorrhoeae* in women under 25 years old annually, especially in all pregnant women under 25 and elderly pregnant women at high risk during their first prenatal visit (13). Furthermore, MSM, people living with HIV/AIDS, and individuals taking HIV pre-exposure prophylaxis (PrEP) are recommended to undergo *C. trachomatis* and *N. gonorrhoeae* screening every 3–6 months for genital, rectal, and pharyngeal infections (13).

Previous studies have demonstrated that intervention strategies, such as promoting STI screening, testing, and treatment among sexually active individuals in the community, providing affordable contraceptive supplies, and implementing sexual health education programs can reduce the incidence of *C. trachomatis* and *N. gonorrhoeae* infections, as well as the prevalence of PID (14, 15, 30). Therefore, the WHO has issued new guidance stating that timely detection of *C. trachomatis* and *N. gonorrhoeae* can significantly reduce the incidence of related complications (https://www.who.int/news/item/24-07-2023-who-releases-new-guidance-to-improve-testing-and-diagnosis-of-sexually-transmitted-infections). The development of simple, rapid, and sensitive POCT assays is a priority, and robust “test-and-treat” capability is essential for optimizing therapeutic interventions, reducing the risk of adverse reproductive outcomes, and curbing hidden community transmission.

Infection with one STI pathogen is known to increase the risk of coinfection with others (31). A previous study reported eight specimens tested positive for both *C. trachomatis* and *N. gonorrhoeae* in 80 cervical and vaginal swabs from women patients in Zhejiang, China (32). In this study, we detected 7 cases with coinfection of *C. trachomatis* and *N. gonorrhoeae* among 156 paired urine and genital secretion samples, corresponding to a coinfection rate of 4.5% (7/156). The difference in coinfection rate may be related to sex difference since the vast majority (93.6%) of patients in this study were male. Large-scale epidemiological investigation on the prevalence of both pathogens is needed. The co-circulation of *C. trachomatis* and *N. gonorrhoeae*, along with a considerable rate of coinfection, underscores the complex epidemiological landscape of STIs, which highlights the needs of large-scale epidemiological investigation on the prevalence of both pathogens, as well as rapid and sensitive multiplex POCT assays.

Although culture had long been regarded as the gold standard for *C. trachomatis* and *N. gonorrhoeae* diagnosis, its practical utility is limited by stringent conditions for culture and transport, lengthy turnaround time, and notably low sensitivity (66.7%–71.9% for *N. gonorrhoeae* and 36.1%–45.7% for *C. trachomatis*) (33, 34). Serological tests for *C. trachomatis* and *N. gonorrhoeae* frequently exhibit limited diagnostic accuracy, increasing the risks of both misdiagnosis and underdiagnosis (33).

Several singleplex and duplex qPCR assays have been approved by the U.S. Food and Drug Administration (FDA) for the detection of *C. trachomatis* and *N. gonorrhoeae* (35), including the Roche Cobas, Abbott Real Time, and GeneXpert *C. trachomatis*/*N. gonorrhoeae* assays. These assays have been evaluated in clinical samples, demonstrating their effectiveness in detecting these pathogens with excellent sensitivity and specificity (36–39). However, strict reliance on the expensive thermal cyclers, specialized laboratory infrastructure, and relatively long turnaround time (about 1.5 h) limit their accessibility in low- and middle-income countries. In this study, we developed HiFi-LAMP assays for the detection of *C. trachomatis* and *N. gonorrhoeae*. The assays exhibited high specificity and sensitivity with LOD of 358 and 30 copies per 25 μL reaction for *C. trachomatis* and *N. gonorrhoeae*, respectively. Clinical evaluation demonstrated well performance of the HiFi-LAMP assays with 100% specificity and 88.5%–100% sensitivity for *C. trachomatis* and *N. gonorrhoeae* in urine and genital secretion samples. In particular, the duplex HiFi-LAMP assay showed 100% concordance with the singleplex assays for 86 clinical samples including 7 coinfected samples.

Genital secretions were found to have slightly higher detection rates of *C. trachomatis* and *N. gonorrhoeae* than urines. The main reason is that both *C. trachomatis* and *N. gonorrhoeae* colonize the epithelial tissue of the genital tract (40, 41), and genital secretions have higher bacterial load than urines. However, compared with genital secretions, urine samples are relatively more patient-friendly since its non-invasive and self-sampling characteristics not only reduce the burden on healthcare providers but also preserve patient privacy, thereby lowering the barriers to routine screening in resource-limited settings (42). Therefore, we developed extraction-free HiFi-LAMP assays for POCT diagnosis of *C. trachomatis* and/or *N. gonorrhoeae* in urines.

Nucleic acid extraction typically requires complex operational procedures and relatively long processing time, limiting its practical application in POCT diagnosis of various infectious diseases. Our previous studies demonstrated that the HiFi-LAMP method has excellent compatibility across various clinical samples, including blood, nasopharyngeal swab, urine, and saliva samples, and can be developed into extraction-free format to detect different pathogens directly using simply treated clinical samples (25, 43, 44). In this study, we established a urine extraction-free HiFi-LAMP assay by simple treatment of urine utilizing a commercial nucleic acid release buffer. Only a very small amount of raw urine (about 0.214 μL) is required to achieve optimal detection performance. Clinical evaluation with 70 urine samples showed that the extraction-free HiFi-LAMP assay had 87.9% sensitivity and 100% specificity for *N. gonorrhoeae* detection. Although the sensitivity was slightly lower than the traditional assay with purified DNA, the assay demonstrated high diagnostic concordance and significantly reduced sample processing and nucleic acid extraction time. Given the non-invasive nature of urine samples, the extraction-free HiFi-LAMP assay provides a feasible and efficient solution for rapid and accurate detection of *C. trachomatis* and *N. gonorrhoeae*, particularly in resource-limited settings where laboratory infrastructures are lacking.

The WHO recommended that POCT products for the detection of *C. trachomatis* and *N. gonorrhoeae* should achieve a sensitivity and specificity greater than 90% and 98%, for *C. trachomatis*, respectively, and both greater than 90% for *N. gonorrhoeae* (https://www.who.int/publications/i/item/9789240077102). The standard HiFi-LAMP assay met these criteria for the detection of *C. trachomatis* in genital secretions and *N. gonorrhoeae* in both urine and genital secretion samples. A slightly lower sensitivity of 88.5% was observed for detection of *C. trachomatis* in urines by the HiFi-LAMP assay, which may be attributed to lower bacterial load in urine samples.

Despite this limitation, the HiFi-LAMP assay offers several advantages, including compatibility with portable devices, simplicity with non-invasive urine samples, low cost, and rapid detection (Table S4). For example, compared to the existing platforms like GeneXpert, our assays are highly cost-effective at below $5 per reaction and have a very short turnaround time (within 40 min). Furthermore, the HiFi-LAMP assays have the potential to meet the WHO “REASSURED” criteria of real-time connectivity, ease of specimen collection, affordable, sensitive, specific, user-friendly, rapid and robust, equipment-free, and deliverable to end-users after further optimization and improvement (45).

### Conclusions

We developed a simple, rapid, sensitive, and highly specific duplex HiFi-LAMP assay for simultaneous detection of *C. trachomatis* and *N. gonorrhoeae*. The LOD of the assays were 358 copies and 30 copies per 25 μL reaction for *C. trachomatis* and *N. gonorrhoeae*, respectively. The method demonstrated excellent diagnostic performance in the detection of *C. trachomatis* and *N. gonorrhoeae* in both urine and genital secretion samples, with 88.5%–100% sensitivity and 100% specificity for 156 paired urine and genital secretion samples. An extraction-free HiFi-LAMP assay directly using simply treated raw urine was developed, showing 87.9% sensitivity and 100% specificity for the detection of *N. gonorrhoeae* in 70 clinical samples. Given the development of various portable real-time fluorescent PCR instruments, the novel HiFi-LAMP assays show high potential for large-scale screening and rapid, POC detection of *C. trachomatis* and *N. gonorrhoeae*, particularly in resource-limited settings.

## Data Availability

All data generated or analyzed during this study are included in this published article and its supplemental material.

## SUPPLEMENTAL MATERIAL

Supplemental material spectrum.03416-25-s0001.docx Tables S1 to S4; Fig. S1 to S5.

ASM does not own the copyrights to Supplemental Material that may be linked to, or accessed through, an article. The authors have granted ASM a non-exclusive, world-wide license to publish the Supplemental Material files. Please contact the corresponding author directly for reuse.
