# Supplementary material for: Duplex, extraction-free, HiFi-LAMP assay for point-of-care detection of Chlamydia trachomatis and Neisseria gonorrhoeae
Source: Microbiol Spectr. 2026 Jul 24;14(9):e03416-25. doi: 10.1128/spectrum.03416-25 (PMC13532307; doi:10.1128/spectrum.03416-25)
Supplement: Supplementary file 1 [file spectrum.03416-25-s0001.docx]

**Supplemental Materials**

**Duplex, extraction-free, HiFi-LAMP assay for point-of-care detection of *Chlamydia trachomatis* and *Neisseria gonorrhoeae***

Zhengfang Wang^1^, Yongjuan Zhao^2^, Yuhua Shi^3^, Shangwen Song^1^, Yingkui Cao^1^, Yumeng Pan^1^, Yang Yang^1^, Yi-Qun Kuang^4^, Chiyu Zhang^2^, Yu-Ye Li^1^

^1^ Department of Dermatology and Venereology, First Aﬃliated Hospital of Kunming Medical University, Kunming, Yunnan, People's Republic of China

^2^ Shanghai Public Health Clinical Center, Fudan University, Shanghai, People's Republic of China

^3^ Yunnan Center for Disease Control and Prevention, Kunming, Yunnan, People's Republic of China

^4^ Research Center for Clinical Medicine, First Aﬃliated Hospital of Kunming Medical University, Kunming, Yunnan, People's Republic of China

Zhengfang Wang, Yongjuan Zhao and Yuhua Shi contributed equally to this work. The author order was determined by their contributions to the writing of the paper.

Corresponding authors: Yi-Qun Kuang ([yq610433@hotmail.com](mailto:yq610433@hotmail.com)), Chiyu Zhang ([chiyu_zhang1999@163.com](mailto:chiyu_zhang1999@163.com)), Yu-Ye Li ([yyeli2000@126.com](mailto:yyeli2000@126.com))

**TABLE S1 Primer sets of the LAMP assays for detection of *C. trachomatis* and *N. gonorrhoeae*.**

| **Primer sets** | **Target genes** | **Primers** | **Sequence (5’→3’)** | **Ref.** |
| --- | --- | --- | --- | --- |
| CT1 | *ompA* | F3 | GAACAGAWGCWGCGACAG | (1) |
|  |  | B3 | YGGGTTTAGAGTAGTRAYATC |  |
|  |  | FIP | TTAACTCCAATGTARGGAGTGAACAATGCCTCTATTGAYTACCAT |  |
|  |  | BIP | GGTCTMGAGYAAGTTTTGATGCCGCAAGATWGCTTYAGCCAATT |  |
|  |  | LF | GARAGRGCTAAACTTGCTTGCC |  |
|  |  | LB | ATYCGTATAGCTCAGCC |  |
| CT2 | *ompA* | F3 | GTWTTTGCCGCTTTGAGTTCTG | (2) |
|  |  | B3 | AAACRCGGTCGAAAACAAAGTC |  |
|  |  | FIP | ATTCCGTCGATCATAAGGCTTGGTCTTCCTCCTTGCAAGCTCTG |  |
|  |  | BIP | TGGGAAGGTTTYGGYGGAGATWCCRTAGTAACCMAYRCGCATG |  |
|  |  | LF | CAGCAGGATTCCCCACA |  |
|  |  | LB | ATCCTTGCRCCACTTGGTG |  |
|  |  | LB1 | TGCGATCCTTGCACCAC | This study |
|  |  | LB2 | ACCACTTGGTGTGACGCTAT |  |
|  |  | HFman-LF* | BHQ1-CAGCAGGATTCCCCACA-FAM |  |
|  |  | HFman-LB** | BHQ1-ATCCTTGCRCCACTTGGTG-FAM |  |
|  |  | HFman-LB1** | BHQ1-TGCGATCCTTGCACCAC-FAM |  |
|  |  | HFman-LB2 ** | BHQ1-ACCACTTGGTGTGACGCTAT-FAM |  |
| CT3 | *gyrA* | B3 | AACACCCTTCTTGGTAGC | (3) |
|  |  | FIP | ACGTTCTCCTTCAGGAAGCTGGATCTTCACCAACTTCGGT |  |
|  |  | BIP | TCCTGGAAGGAATCCGTCCCGGTATTCTCCTTGCTCGAAC |  |
|  |  | LB | GAGAACAAGTTGCTGCGGTGCT |  |
| NG1 | *penA* | F3 | TGACCAGTAACGGACGGATT | (4) |
|  |  | B3 | CTCAACGCCAAAACCGCA |  |
|  |  | FIP | TACCGTCTGCAGATACAGCCCGAATGGCGGTCTTGTTTTCCT |  |
|  |  | BIP | TGAAAGAACAGGGCGACAACCGGCGTGTAGCCGGCAGTGC |  |
|  |  | LF | CGGGCAATCAGACAGGCAA |  |
|  |  | LB | GATTGTGCGGACTCAAGCAT |  |
| NG2 | *orf1* | F3 | CCGCGTATTTTGCCATTTCC | (5) |
|  |  | B3 | ACCGTTTTCGAACCACTCTT |  |
|  |  | FIP | AATCGACAAAGCGGGCTTTGCTTCAGGAGCTGAAGATGTCCA |  |
|  |  | BIP | TCACCGATACCAAAGGCAAGCAGGGCCTTCCAACACGATG |  |
|  |  | LF | TCAATCAATTTTACGGCG |  |
|  |  | LB | ACCACTTTACCGTGCCTG |  |
|  |  | LB1 | ACTTTACCGTGCCTGCG | This study |
|  |  | LF1 | TTTCAATCAATTTTACGGCGTT |  |
|  |  | LB2 | GCACCACTTTACCGTGCCTG |  |
|  |  | LB3 | CTTTACCGTGCCTGCGCG |  |
|  |  | LF2 | TTTTTCAATCAATTTTACGGCGTTT |  |
|  |  | HFman-LF* | BHQ2-TCAATCAATTTTACGGCG-CY5 |  |
|  |  | HFman-LB** | BHQ2-ACCACTTTACCGTGCCTG-CY5 |  |
|  |  | HFman-LB1** | BHQ2-ACTTTACCGTGCCTGCG-CY5 |  |
|  |  | HFman-LF1** | BHQ2-TTTCAATCAATTTTACGGCGTT-CY5 |  |
|  |  | HFman-LB2** | BHQ2-GCACCACTTTACCGTGCCTG-CY5 |  |
|  |  | HFman-LB3** | BHQ2-CTTTACCGTGCCTGCGCG-CY5 |  |
|  |  | HFman-LF2** | BHQ2-TTTTTCAATCAATTTTACGGCGTTT-CY5 |  |
| NG3 | *porA* | F3 | CCATTGATCCTTGGGACAG | (6) |
|  |  | B3 | CAGACCGGCATAATACACAT |  |
|  |  | FIP | GGGAATCGTAACGCACGGAAATAATGTGGCTTCGCAATTG |  |
|  |  | BIP | AGCGGCAGCATTCAATTTGTTCCTGATTACTTTCCAGCGTGA |  |
|  |  | LF | ATACCGTCGTGGCGTTTG |  |
|  |  | LB | CGCCTATACGCCTGCTAC |  |

Note: * The original probe; ** optimized probe; CT, *C. trachomatis*; NG, *N. gonorrhoeae*.

**TABLE S2 Demographic characteristics of 156 patients with paired urine and genital secretion samples.**

| **Characteristic category** | | **N (%)** |
| --- | --- | --- |
| Gender | Male | 146 (93.6%) |
|  | Female | 8 (5.1%) |
|  | Unknown | 2 (1.3%) |
| Age | years (Mean±SD) | 32.80±12.18 |
|  | Range of age (years old) | 15-71 |
|  | ≤19 | 16 (10.3%) |
|  | 20-29 | 51 (32.7%) |
|  | 30-39 | 46 (29.5%) |
|  | 40-49 | 15 (9.6%) |
|  | 50-59 | 13 (8.3%) |
|  | ≥60 | 5 (3.2%) |
|  | Unknown | 10 (6.4%) |
| Education level | Illiterate | 3 (1.9%) |
|  | Primary school | 11 (7.1%) |
|  | Junior middle school | 28 (17.9%) |
|  | Senior high school | 18 (11.5%) |
|  | Bachelor's degree | 72 (46.2%) |
|  | Master's degree | 5 (3.2%) |
|  | Unknown | 19 (12.2%) |
| Marital status | Unmarried | 66 (42.3%) |
|  | Married | 67 (43.0%) |
|  | Divorced | 3 (1.9%) |
|  | Unknown | 20 (12.8%) |

**TABLE S3** **Comparison of the duplex HiFi-LAMP and singleplex HiFi-LAMP assays for detection of *C. trachomatis* and *N. gonorrhoeae* in 86 urine samples.**

| **Methods** | | **Duplex HiFi-LAMP assay** | | | | |
| --- | --- | --- | --- | --- | --- | --- |
| Singleplex HiFi-LAMP assays |  | CT | NG | CT and NG coinfection | Neg. | Total |
|  | CT | 13 | 0 | 0 | 0 | 13 |
|  | NG | 0 | 59 | 0 | 0 | 59 |
|  | CT and NG coinfection | 0 | 0 | 7 | 0 | 7 |
|  | Neg. | 0 | 0 | 0 | 7 | 7 |
|  | Total | 13 | 59 | 7 | 7 | 86 |

Note: CT: *C. trachomatis*; NG: *N. gonorrhoeae*; Neg. negative for both pathogens.

**TABLE S4** **Feature comparison of the HiFi-LAMP assay with** **three commercial assays.**

| Features | HiFi-LAMP (This Study) | GeneXpert (Cepheid) | Binx io (Binx Health) | Visby Medical Women's Sexual Health Test (Visby Medica) |
| --- | --- | --- | --- | --- |
| Pathogen Detection | CT/NG | CT/NG | CT/NG | CT/NG/TV |
| Sample Type | urine, genital swab | urine, swabs | urine, swabs | vaginal swab |
| Turnaround Time | about 30 min | about 90 min | about 30 min | about 28 min |
| Cost | Low (about $0.5/test) | High ($17–$35/test) | High ($70+) | High ($120/test) |
| Equipment | portable real-time device (low cost: about $1,461) | Large Platform (expensive: about $70,000) | Large Platform (expensive: about $13,000) | None (single-use device) |

Note: CT: *C. trachomatis*; NG: *N. gonorrhoeae*; TV: *Trichomonas vaginalis*.

**FIG S1 Screening of optimal LAMP primer sets for detection of *C. trachomatis* and *N. gonorrhoeae*.** (**A**) Performance of three primer sets (CT1, CT2, CT3) for CT detection. (**B**) Performance of three primer sets (NG1, NG2, NG3) for NG detection. CT: *C. trachomatis*; NG: *N. gonorrhoeae*.

**FIG S2 Sequence analysis of loop primers of the primer sets CT1 and CT2**. CT: *C. trachomatis*.

**FIG S3 Optimization of the primer sets of the HiFi-LAMP assays for detection of *C. trachomatis* and *N. gonorrhoeae*.** (**A**) Primer set CT2 NTC. (**B**) Primer set CT2 NTC. (**C**) Primer set CT2 with different probes. (**D**) Set CT2-LB for NTC. (**E**) Set NG2 with different probes. (**F**) Set NG2-LB1 for NTC. (**G**) Optimization of set NG2 with different probes. (**H**) Set NG2-LB3 for NTC. (**I**) Set NG2 LF1 or LF2 probes with LB1, LB2 or LB3. (**J**) Set NG2-LF2+LB1 for NTC. (**K**) Set NG2-LF2+LB3 for NTC. (**L**) Set NG2-LF1+LB2 for NTC. Pos., positive. NTC, non-template control; CT, *C. trachomatis*; NG, *N. gonorrhoeae*.

**FIG S4 Comparison of the qPCR Ct values between positive urine and genital secretion samples.** (A) Detection of *C. trachomatis*. (B) Detection of *N. gonorrhoeae*.

**FIG S5 Detection of co-infected samples by the duplex HiFi-LAMP assay.** “Note: +”, positive result for NG or CT; CT: *C. trachomatis*; NG: *N. gonorrhoeae*.

**Reference**

1.	Choopara I, Arunrut N, Kiatpathomchai W, Dean D, Somboonna N. 2017. Rapid and visual Chlamydia trachomatis detection using loop-mediated isothermal amplification and hydroxynaphthol blue. Lett Appl Microbiol 64:51-56.<https://doi.org/10.1111/lam.12675>

2.	Chen X, Zhou Q, Tan Y, Wang R, Wu X, Liu J, Liu R, Wang S, Dong S. 2022. Nanoparticle-Based Lateral Flow Biosensor Integrated With Loop-Mediated Isothermal Amplification for Rapid and Visual Identification of Chlamydia trachomatis for Point-of-Care Use. Front Microbiol 13:914620.<https://doi.org/10.3389/fmicb.2022.914620>

3.	Xu X, Jia Y, Li R, Wen Y, Liang Y, Lao G, Liu X, Zhou W, Liu H, Xie J, Wang X, Xu W, Sun Q. 2022. Rapid and simultaneous detection of multiple pathogens in the lower reproductive tract during pregnancy based on loop-mediated isothermal amplification-microfluidic chip. BMC Microbiol 22:260.<https://doi.org/10.1186/s12866-022-02657-0>

4.	Shimuta K, Nakayama SI, Takahashi H, Ohnishi M. 2019. A Loop-Mediated Isothermal Amplification Assay Targeting Neisseria gonorrhoeae penA-60.001. Antimicrob Agents Chemother 64.<https://doi.org/10.1128/aac.01663-19>

5.	Chen X, Zhou Q, Wu X, Wang S, Liu R, Dong S, Yuan W. 2021. Visual and Rapid Diagnosis of Neisseria gonorrhoeae Using Loop-Mediated Isothermal Amplification Combined With a Polymer Nanoparticle-Based Biosensor in Clinical Application. Front Mol Biosci 8:702134.<https://doi.org/10.3389/fmolb.2021.702134>

6.	Rodriguez-Mateos P, Ngamsom B, Ameyo D, Wakaba P, Shiluli C, Iles A, Gitaka J, Pamme N. 2023. Integrated microscale immiscible phase extraction and isothermal amplification for colorimetric detection of Neisseria gonorrhoeae. Anal Bioanal Chem 415:5129-5137.<https://doi.org/10.1007/s00216-023-04734-3>
